# Analysis of Flavonoids from *Eugenia uniflora* Leaves and Its Protective Effect against Murine Sepsis

**DOI:** 10.1155/2012/623940

**Published:** 2012-12-20

**Authors:** Yanna D. Rattmann, Lauro Mera de Souza, Simone M. Malquevicz-Paiva, Nessana Dartora, Guilherme Lanzi Sassaki, Philip A. J. Gorin, Marcello Iacomini

**Affiliations:** ^1^Departamento de Bioquímica e Biologia Molecular, Universidade Federal do Paraná, CP 19046, 81531-980 Curitiba, PR, Brazil; ^2^Departamento de Saúde Comunitária, Universidade Federal do Paraná, Rua Padre Camargo, 280, Alto da Glória, 80060-240 Curitiba, PR, Brazil

## Abstract

*Eugenia uniflora*, referred to as Pitanga cherry shrub, is largely distributed in tropical and subtropical America. This plant is cultivated in many countries and it is suitable for the production of juice, frozen pulp, and tea. Besides, it can be used as treatment for inflammatory diseases. We reported that a flavonoid-rich fraction (HE-Bu) obtained from leaves decreased the lethality induced by cecal ligation and puncture (CLP), a clinically relevant model of sepsis. The oral administration of HE-Bu reduced the late mortality rate by 30%, prevented neutrophil accumulation in lungs, decreased TNF-**α** and IL-1**β** serum levels, and markedly decreased iNOS and COX-2 protein expression by ileum cells. Chemical investigation showed myricetin and quercetin rhamnosides as the major components of this fraction. The results showed that HE-Bu protected mice from sepsis and indicated that this edible plant produces compounds that could be considered as potential adjuvants for sepsis treatment.

## 1. Introduction

*Eugenia uniflora* L. (Myrtaceae) is a tropical and subtropical shrub widely distributed in American countries [1]. It is commonly referred to as Pitanga cherry or Brazilian cherry. Regarding their effects on human health, both fruit and leaves are used as folk medicine to treat similar diseases, although the leaves show the advantage of being perennial and continuously available, while the fruit are available during a short period of the year [2]. The fresh or dried leaves have been used empirically as medicine, since the 15th century [3], for treating inflammatory and stomach diseases, rheumatism, fever, and hypertension [4, 5]. Some studies have confirmed that *Eugenia uniflora* possesses anti-inflammatory, antimicrobial, and antifungal properties [4, 6–8]. These benefits are usually attributed to the presence of many secondary metabolites present in the leaves, which includes many volatile terpenoid oils, flavonoids, and condensed and hydrolysable tannins, leucoanthocyanidins, and steroids and/or triterpenoids [9].

Flavonoids are presented in many plant extracts, being constantly the focus of pharmacological studies. Despite of their well-described antioxidant activity [10–12], they have shown many other properties, such as anti-inflammatory, antimicrobial, antiaging, anticancer, and antiallergic, hypocholesterolemic and vasodilatation, [10–15]. Together, the anti-inflammatory and antioxidant properties of flavonoids can explain the efficacy of plant extracts against various diseases, such as osteoporosis and rheumatism [15]. Therapeutic properties attributed to the flavonoid contents of *E. uniflora* against inflammatory disorders were reported, but not dealing with the ability of this plant in reducing the mortality caused by experimental sepsis, which is closely related to inflammation [16].

Sepsis is an important health problem and a leading cause of morbidity and mortality in many intensive care units. This condition is characterized by the overproduction of proinflammatory mediators, which frequently occurs after various noxious injuries, especially bacterial infection, as a consequence of abdominal surgery, appendicitis, perforated ulcers, or an ischemic bowel [17].

A common cause of sepsis is the exposure to lipopolysaccharide (LPS), the structural component of gram-negative bacteria membrane, or to peptidoglycan and lipoteichoic acid, structural components of gram-positive bacteria membrane [17]. The main clinical signs include fever, hypotension, vasoplegia, and neutrophil infiltration, which may lead to multiple organ dysfunction and ultimately death. The bacterial components in the bloodstream induces the overexpression of various inflammatory mediators by the immune system cells, such as interleukin-1*β* (IL-1*β*), tumor necrosis factor-*α* (TNF-*α*), interleukin-6 (IL-6), interleukin-8 (IL-8), nitric oxide (NO), and prostanoids. This large amount of inflammatory mediators is thought to contribute to septic shock and mortality [18]. 

Thus, phytochemical analysis of a flavonoid-rich fraction obtained from *Eugenia uniflora* leaves was performed and the biological properties of this fraction, such as its effects on lethality, neutrophil migration, cytokine levels, and iNOS and COX-2 tissue expression, were evaluated using a murine sepsis model. 

## 2. Material and Methods

### 2.1. Plant Material

Fresh leaves of *Eugenia uniflora* were harvested in the Campus Centro Politécnico of the Federal University of Paraná, Curitiba, PR, Brazil. The plant was identified according to an authentic sample deposited at the Department of Botany of the same university, where a voucher specimen is deposited.

### 2.2. Sample Preparation

#### 2.2.1. Extraction and Fractionation of Compounds from Leaves

Fresh leaves of *E. uniflora* (100 g) were twice extracted in 300 mL of *n*-hexane under reflux. The extracts were combined, dried at room temperature (~22°C), and stored in at −20°C. The leaves residue was submitted to hydroalcoholic extraction, in 300 mL of ethanol 70% (v/v), repeated thrice. The extracts were filtered off, combined, and dried under reduced pressure, giving rise to a crude hydroalcoholic extract (HE). 

HE was fractionated by degree of polarity using a liquid-liquid partition as follows: 1 g was dissolved in H_2_O (200 mL), followed by the addition of CHCl_3_ (200 mL) and vigorous stirring. The mixture was left in a decanting funnel until the formation of two phases, which were separated. The CHCl_3_ fraction (HE-Chl) was dried under reduced pressure and stored at −20°C. The aqueous fraction was completed with additional 200 mL of ethyl acetate, followed by the same separation procedure, giving rise to an organic fraction (HE-EA), which was evaporated and stored. Subsequently, *n*-butanol (200 mL) was added to the remaining aqueous layer, mixed, and separated in two new phases: the butanol (HE-Bu) and the aqueous one (HE-Aq), that were evaporated under reduced pressure and stored, separately, at −20°C.

#### 2.2.2. Chemical Derivatization

In order to determine its chemical composition, the fraction HE-Bu (2 mg) was hydrolyzed in 0.5 mL of 45% formic acid (v/v), 14 h, 100°C [19]. The sample was dried under N_2_ stream, then mixed in *n*-butanol-H_2_O (1 : 1, v/v), yielding two layers which were separated and evaporated. The organic layer was evaluated by liquid chromatography analysis, whereas the aqueous one was submitted to successive reduction in NaBH_4_ and acetylation in pyridine-acetic anhydride (200 *μ*L; 1 : 1, v/v) to analyze the monosaccharide composition, by gas chromatography-mass spectrometry (GC-MS) [19].

### 2.3. Analytical Procedures

#### 2.3.1. Ultrahigh Performance Liquid Chromatography-Mass Spectrometry (UHPLC-MS)

This was carried out in an Acquity-UPLC system (Waters, MA, USA), composed by a binary pump, sample manager, and column oven. Detection was provided by a photodiode array detector (PDA) and a triple quadrupole, electrospray ionization-mass spectrometry (ESI-MS) Quattro LC (Wates, MA, USA). The separations were developed on Waters BEH-C18 column, with 50 × 2.1 mm length and 1.7 *μ*m particle size, using as solvent A, formic acid 1% (v/v), and solvent B methanol. The samples were held at room temperature (22°C) and the column oven was kept at 60°C. A gradient of solvent B was used, from 0% to 40%, over 7 min, then to 80% in 10 min, backing to initial condition (100% solvent A) in 11 min, with a flow rate of 400 *μ*L·min^−1^. The sample HE-Bu (1 mg·mL^−1^) was dissolved in MeOH-H_2_O (1 : 1, v/v). The volume for injection was 3 *μ*L and the detection was provided by PDA (200–400 nm) and ESI-MS (*m/z* 100–1100). The ESI-MS energies were set at 2.5 kV on the capillary and 55 V on the cone (negative ion mode). Tandem-MS was obtained by collision induced dissociation-mass spectrometry (CID-MS), using argon as collision gas. The collision energies ranged from 50 to 80 eV.

#### 2.3.2. Gas Chromatography-Mass Spectrometry (GC-MS)

This was carried out in a Varian Saturn 2000 R, equipped with a capillary column DB-225-MS (J&W), 30 m long. The mass spectrometry was consisted of an Ion Trap detector, with electron ionization (EI) operating at 70 eV. Each monosaccharide was previously converted to its alditol acetate and identified using authentic standards.

### 2.4. Animals

Male albino Swiss mice (3 months old, weighing 25–30 g) from the Federal University of Paraná were used for biological tests. They were maintained under standard laboratory conditions, at a 12 h light/dark cycle and controlled temperature (22 ± 2°C). Standard pellet food (Nuvital, Curitiba, PR, Brazil) and water were available *ad libitum*. All experimental procedures were previously approved by the Institutional Ethics Committee of the University (authorization number 430).

### 2.5. Experimental Protocols

#### 2.5.1. Procedure to Induce Sepsis by Cecal Ligation and Puncture (CLP)

Mice were randomly divided into five groups, containing 10 mice/group: sham operation; CLP plus vehicle (water + ethanol 3%; p.o.); CLP plus HE-BU 75 mg·kg^−1^ p.o.; CLP plus HE-Bu 150 mg·kg^−1^ p.o.; CLP plus HE-Bu 300 mg·kg^−1^, p.o. It was administered 30 *μ*L of each treatment solution, regarding the corporeal weigh of the animals (~30 g). Ketamine (80 mg·kg^−1^) and xylazine (20 mg·kg^−1^) were injected intraperitoneally to anesthetize the mice before the surgical procedures. Polymicrobial sepsis was induced by CLP as previously described [20]. A midline incision about ~1.5 cm was performed on the abdomen. The cecum was carefully isolated and the distal 50% was ligated. The cecum was then punctured twice with a sterile 20-gauge needle and squeezed to extrude the fecal material from the wounds. The cecum was replaced and the abdomen was closed in two layers. Sham-control animals were treated identically, but no cecal ligation or puncture was carried out. Each mouse received subcutaneous sterile saline injection (1 mL) for fluid resuscitation after surgery. The mice were then placed on a heating pad until they recover from the anesthesia. Food and water *ad libitum* were provided throughout the experiment. Survival rate was monitored for 7 days, each 12 h. During this period, vehicle (water + 3% ethanol) was orally administered daily. 

For the next experiments, 1 h before the surgery, mice were orally treated with vehicle, HE-Bu (75, 150, or 300 mg·kg^−1^, p.o.), and after 6 h postoperation, all mice were sacrificed. Their tissues from lungs, small intestines (ileum), and blood were collected and frozen for later use to determine the myeloperoxidase (MPO) activity and investigate cytokine production in serum and iNOS and COX-2 tissue expression. Dexamethasone, a corticosteroid anti-inflammatory drug, was used as control and was administered subcutaneously at dose 0.5 mg/kg.

#### 2.5.2. Lung MPO Activity

The MPO activity, assessment of the neutrophil influx, was determined according to established protocols [21], after 6 h postoperation. Briefly, lung tissue was homogenized in 0.5 mL of 50 mM potassium buffer pH 6.0 with 0.5% hexadecyltrimethylammonium bromide, sonicated on ice, and then centrifuged at 14,000 rpm for 15 min at 4°C. Supernatants were diluted (1 : 20) in reaction buffer (9.6 mM 3,3,5,5-tetramethylbenzene, 150 nmol·L^−1^ H_2_O_2_ in 50 mM potassium phosphate buffer) and read at 620 nm. Results are expressed as change in optical density per milligram of protein (measured by the Bradford assay).

#### 2.5.3. Determination of Cytokine Levels

Tumor necrosis factor alpha (TNF-*α*) and interleukin (IL)-1*β* concentrations were determined in mice serum using enzyme linked immunosorbent assay (ELISA) kits (R&D Systems, Minneapolis, MN, USA) according to the manufacturer's instructions.

#### 2.5.4. Western Blot Analysis

The ileum samples were washed twice with PBS and then homogenated and lysed in extraction buffer (composition in mM: Tris/HCl 20 (pH 7.5; QBiogene), NaCl 150, Na_3_VO_4_, sodium pyrophosphate 10, NaF 20, okadaic acid 0.01 (Sigma), a tablet of protease inhibitor (Roche), and 1% Triton X-100 (QBiogen)). Total protein (20 *μ*g) was separated on 8% SDS-polyacrylamide (Sigma) gels at 80 V for 2 h. Isolated proteins were transferred electrophoretically onto polyvinylidene difluoride membranes (Bio-Rad) at 100 V for 120 min. Membranes were blocked with blocking buffer containing 3% low-fat milk powder, Tris-buffered saline solution (Bio-Rad), and 0.1% Tween 20 (Sigma) (TBS-T) for 1 h. Membranes were then incubated with the primary antibodies of either iNOS and COX-2 (dilution of 1 : 1000), overnight at 4°C. After washing, membranes were incubated with the secondary antibody (peroxidase-labeled anti-mouse IgG, dilution of 1 : 5000) at room temperature for 60 min. Detection of *β*-tubulin proteins were used for normalization and quantification of iNOS and COX-2. Prestained markers (Invitrogen) were used for molecular mass determinations. Immunoreactive bands were detected by enhanced chemiluminescence (Bio-Rad).

### 2.6. Statistical Analysis

Data were expressed as means ± SE of five or ten mice examined for each group. Statistical error was determined by one-way ANOVA; the post hoc test was Bonferroni's. Calculations performed with Graphpad Prism 5.0.* P* Values < 0.05 were considered significant. Survival analyses were compared by a logrank test. These calculations were performed with SigmaStat v3.10 (Systat Software Inc, Richmond, CA, USA). The null hypothesis was rejected when  *P* < 0.05.

## 3. Results and Discussion

*Eugenia uniflora* has been known to present a variety of beneficial effects including antimicrobial, antifungal, antipyretic; antioxidant, anti-inflammatory, and antinociceptive activities [4, 6–8]. However, there are no reports regarding the ability of *E. uniflora* of reducing the experimental sepsis outcome, despite its anti-inflammatory and antimicrobial activity. In this study, a polymicrobial sepsis was induced by cecal ligation and puncture (CLP) in mice to investigate the effects of a flavonoid-rich fraction obtained from *Eugenia uniflora* leaves (HE-Bu). This CLP model mimics sepsis in human, caused by pathogens derived from the intestinal tract, and it is considered closely related to the clinical situation [17]. 

**Figure 1:**
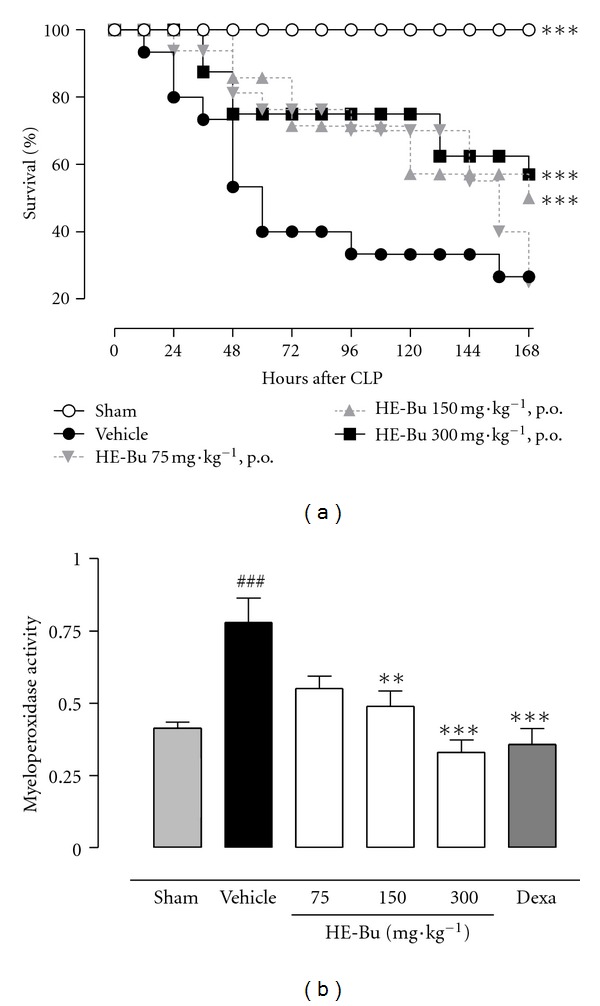
HE-Bu obtained from *Eugenia uniflora* leaves protects against sepsis-induced lethality and inhibits myeloperoxidase activity (measured after 6 h postoperation). Mice (10 animals/group) were orally administered various doses of HE-Bu (75, 150 or 300 mg·kg^−1^), vehicle (3% ethanol), or dexamethasone (0.5 mg·kg^−1^ s.c.). MPO graph: values represent means ± SEM. ***P < 0.001, and **P < 0.01, indicated value versus CLP plus vehicle group; ^###^ *P* < 0.001, CLP plus vehicle versus sham. ANOVA followed by Bonferroni's test. Survival graph: survival analyses were compared by a logrank test. These calculations were performed with SigmaStat v3.10 (Systat Software Inc, Richmond, CA, USA). The null hypothesis was rejected when *P* < 0.05.

It was observed that lethality was markedly delayed in mice orally administered with HE-Bu (see Figure 1(a)). Mice treated with vehicle started to die between 12 h and 24 h after CLP, with a death rate reaching 26.7% and 60.0% after 36 h and 96 h after CLP, respectively. The overall mortality in this group, at the end of the observation period, was 73.3%, and the corresponding area under the curve was 8.117 (arbitrary units). The lethality was markedly delayed in mice treated orally with HE-Bu. Their areas under the curve were increased to 12.420, 12.726, and 12.492 after treating with HE-Bu 75, 150, and 300 mg·kg^−1^, respectively. At the end of the study, the overall survival in these HE-Bu groups was 25%, 50%, and 57%. No death occurred in the sham-operated mice and its corresponding value for area under the lethality curve was 16.800 (arbitrary units). This result could be attributable to an anti-inflammatory activity, which is consistent with previous literature findings [5]. 

It is believed that sepsis may lead to aberrant host inflammatory responses, causing cell injury and organ dysfunction. Neutrophil infiltration is an important pathophysiologic alteration associated with sepsis. These cells cause directly damage to the tissues by releasing proinflammatory mediators, as cytokines, superoxide-derived free radicals, and lysosomal enzymes, such as MPO, which amplify the systemic inflammatory response and cause multiple organ failure [22].

Considering that MPO is a lysosomal enzyme, produced by polymorphonuclear leukocytes, and related to the production of hypochlorous acid (powerful oxidant), the effects of HE-Bu treatment on MPO activity were also investigated. CLP surgery markedly increased lung tissue MPO levels compared with sham group (46.9%) (Figure 1(b)). This increase in tissue MPO was significantly prevented by HE-Bu 150 and 300 mg/kg, with an inhibition of 37.2% and 57.6%, respectively, versus vehicle group (Figure 1(b)). Dexamethasone, the anti-inflammatory drug used as control, strongly inhibited the MPO activity in lungs (54.2%). Therefore, HE-Bu prevented the elevation of MPO activity, indirectly indicating reductions in neutrophil recruitment to ileum, and also in oxidative tissue damage (Figure 1(b)). This is of particular relevance because oxidative stress is known as a probable mechanism for gut mucosal barrier dysfunction during sepsis condition, amplifying and perpetuating the initial systemic inflammatory responses [23].

**Figure 2:**
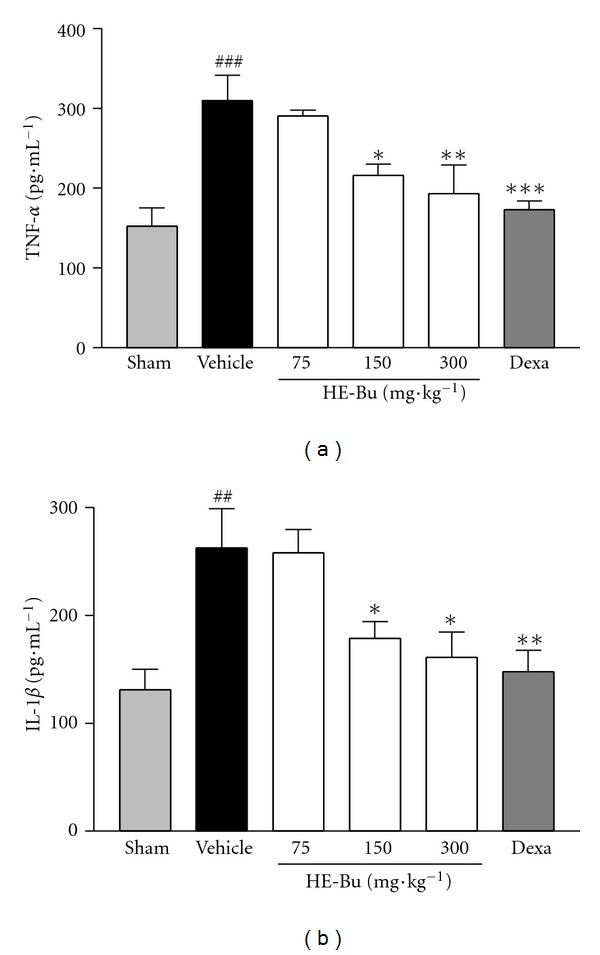
Effect of HE-Bu obtained from *Eugenia uniflora* upon TNF-*α* (a) and IL-1*β* (b) production in infected mice serum. The mice, except the sham group, received vehicle, HE-Bu 75, 150. and 300 mg·kg^−1^ (p.o.) or dexamethasone (0.5 mg·kg^−1^ s.c.), which were administered 1 h before CLP surgery, and the cytokines levels were evaluated 6 h after onset. Each group represents the mean ± SEM of four to five animals. *P < 0.05, **P < 0.01, and ***P < 0.001 indicated value versus CLP plus vehicle group; ^##^ *P* < 0.01 and ^###^ *P* < 0.001, CLP plus vehicle versus sham. ANOVA followed by Bonferroni's test. A false-operated group (sham) was also provided for this test.

 Several previous studies have demonstrated that some cytokines, especially TNF-*α* and IL-1*β* are strongly associated with sepsis syndrome; therefore, inhibiting the proinflammatory cytokine overproduction during early sepsis may reduce its risks [17]. In this study, the HE-Bu was able to decrease significantly TNF-*α* and also IL-1*β* serum levels (Figures 2(a) and 2(b)), and it may be related to the mortality reduction.

TNF-*α* and IL-1*β* cytokines levels were lower in sham surgery control mice. In contrast, 6 h after CLP surgery, both cytokine showed an increase of 50.8% and 50.2%, respectively, in comparison with the sham group (Figures 2(a) and 2(b)). The treatment with HE-Bu showed significant reduction in cytokine production in relation to saline group. HE-Bu 150 and 300 mg·kg^−1^ reduced the levels of TNF-*α* by 33.6% and 38.8%, and the levels of IL-1*β* were also inhibited by 32.3% and 38.5%, respectively. The increased levels of TNF-*α* and IL-1*β* were significantly prevented by dexamethasone (0.5 mg·kg^−1^), approximately 44% of inhibition in both cases, in comparison with the vehicle group (Figures 2(a) and 2(b)).

Besides the cytokines, the nitric oxide (NO) and eicosanoids are also mediators involved in the excessive proinflammatory response during sepsis. Overproduction of NO by inducible nitric oxide synthase (iNOS) is associated with the septic shock, considered as the main cause of mortality among the septic patients [22]. The iNOS is induced in response to inflammatory stimuli such as bacterial lipopolysaccharide (LPS) and proinflammatory cytokines (e.g., IL-1, TNF-*α*). Once expressed, iNOS produces high amounts of NO over long periods of time, which causes cellular damage. Compounds that inhibit iNOS expression or iNOS activity have anti-inflammatory properties, on the basis of their effects in various forms of experimentally induced inflammation [24]. 

Another important product of inflammation from cells of the innate immune system is cyclooxygenase-2 (COX-2). This inducible isoform of the cyclooxygenase enzyme catalyzes the formation of inflammatory prostanoids, such as prostaglandins and thromboxane, which can mediate a significant inflammatory response. Systemic COX-2 is increasingly recognized as an important player in sepsis-induced inflammation. In fact, COX-2-deficient mice are protected from sepsis-induced inflammation and death [25]. 

**Figure 3:**
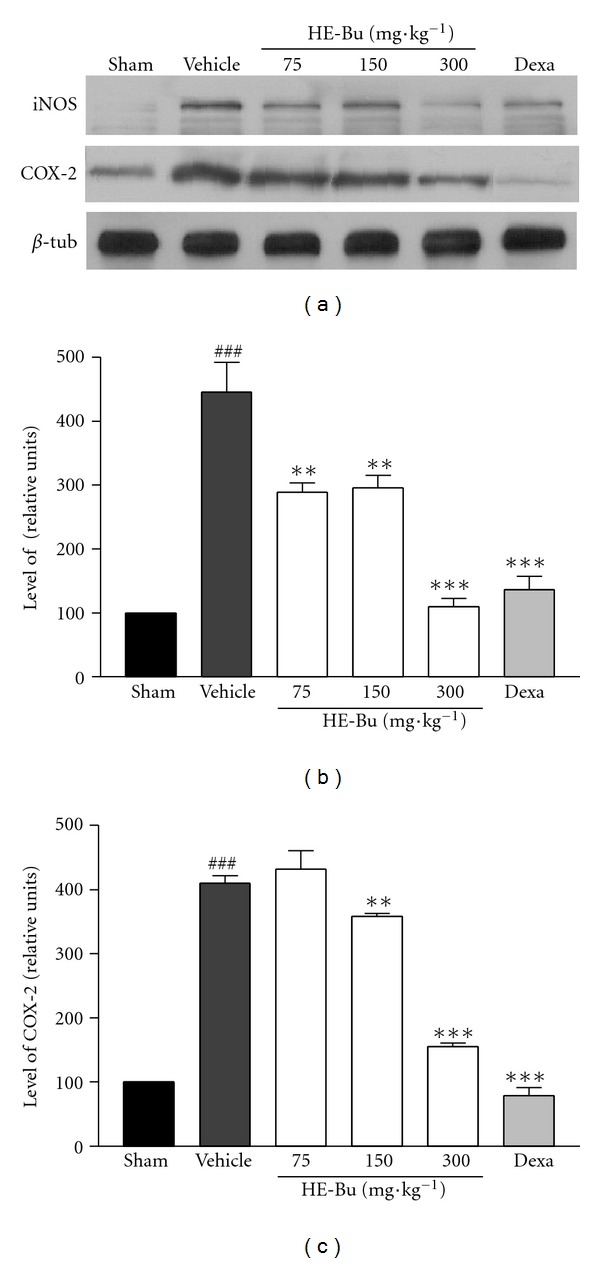
HE-Bu from *E. uniflora* inhibits iNOS (b) and COX-2 (c) expression in ileum of infected mice. Mice were treated with HE-Bu 75, 150, or 300 mg·kg^−1^, p.o. or dexamethasone. The levels of iNOS and COX-2 were determined by western blot analysis. Representative immunoblots (a). Results are shown as the means ± SEM of 2 to 3 different experiments. ^###^ *P* < 0.001, CLP plus vehicle versus sham. **P < 0.01 and ***P < 0.001, HE-Bu versus vehicle.

 In this study, we examined the effects of HE-Bu, by immunoblotting, on iNOS and COX-2 expression by ileum cells of septic mice. HE-Bu (75, 150, and 300 mg·kg^−1^) decreased the levels of iNOS by 35.2%, 33.5%, and 75.5% respectively (Figures 3(a) and 3(b)). The COX-2 expression was reduced by 12.7, and 62% after treating the animals with the higher doses of HE-Bu. Dexamethasone significantly affected both iNOS and COX-2 expression, reducing by 69.5% and 80.8%, respectively (Figures 3(a) and 3(c)). These results clearly indicated that HE-Bu is able to decrease the levels of both investigated proinflammatory enzymes (iNOS and COX-2) whose role is increasingly recognized in the pathophysiology of sepsis.

**Figure 4:**
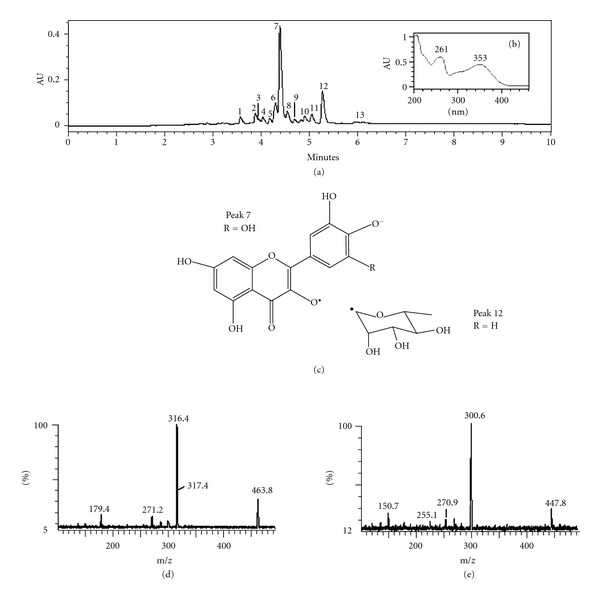
(a) Chromatogram of fraction HE-Bu detected by PDA 360 nm. All peaks had absorbance curves characteristic of flavonols (b). (c) Schematic representation of the homolytic cleavage on CID-MS of peaks 7 (d) and 12 (e) identified as myricetin and quercetin rhamnosides, respectively.

Considering that the overwhelming inflammatory and immune responses during the early stage of sepsis involve a vast array of mediators, it is critical to control this complex inflammatory cascade and consequently to manage the sepsis effects [17]. Thus, natural products containing various components that act in different inflammatory cascades may have an advantage on sepsis treatment when administered in association with conventional drugs which target a single mediator. In this study, many flavonoids were found in the HE-Bu from *E. uniflora* leaves, consisted mainly by glycosides of quercetin and myricetin, by using the offline ESI-MS (Figure 4 and Table 1). 

 With the aim of identifying more compounds, HE-Bu was acid hydrolyzed, followed by a liquid-liquid partition. The organic layer was analyzed by UHPLC-PDA-MS, which confirmed the presence of quercetin and myricetin, by comparison with authentic material (Sigma). The monosaccharides present in the aqueous phase were analyzed by GC-MS as their alditol acetates, showing the presence of arabinose (20%), rhamnose (28%), galactose (21%), and glucose (31%). Considering the absence of other monosaccharides, the presence of arabinose and rhamnose on the flavonoids could be stated without any misinterpretation, whereas galactose and glucose could not. As stated above, the glycosylation site on flavonols occurring in fruit of *E. uniflora* is the 3-*O* position and probably those occurring in leaves follow a similar pattern [26].

 Then, HE-Bu was submitted to UHPLC-PDA-MS analysis obtained in the reversed phase (Figure 1(a)). On PDA, all detected peaks showed *λ*_max⁡_ at 350–360 nm (Band I) and ~255 nm (Band II), characteristic of flavonols (Figure 4(b)) [27]. The chromatogram contained two main peaks, being peak 7 identified as myricetin-rhamnoside by the formation of a deprotonated ion at *m/z* 463 [M-H]^−^, and a main fragment at *m/z* 316 resulting from homolytic cleavage occurring in the glycosidic linkage, yielding a radical ion from the flavonol core (Figures 4(b) and 4(c)) [28]. Similarly, the second main peak (12) appeared at *m/z* 447, with a main fragment at *m/z* 300, consistent with a quercetin-rhamnoside (Figure 1(c)). This marked formation of radical fragments from aglycones also suggests the 3-*O-*glycosylation site [29, 30].

Compounds of low abundance were consistent with other glycosides of quercetin and myricetin, containing different monosaccharides (Table 1). Nevertheless, four of them appeared attached by an additional gallic acid moiety, yielding deprotonated ions at *m/z* 631 (peak 1), consistent with myricetin-hexosyl-gallate, *m/z* 615 (peaks 4 and 5) with quercetin-hexosyl-gallate, and *m/z* 599 (peak 8) with quercetin-rhamnosyl-gallate. With the exception of peak **8**, this type of glycoside has been previously reported in other plants, as quercetin 3-*O*-glucosyl-6′′-*O*-gallate, found in *Tellima grandiflora* and *Polygonum hydropiper* L. [31, 32] and recently in *Camellia sinensis* [33].

It has been reported that quercetin attenuates lethal systemic inflammation caused by endotoxemia, even if treatment is started after the early TNF response, and also that quercetin is able to inhibit the iNOS and COX-2 gene expressions in macrophages [34]. It was shown that myricetin has an anti-inflammatory property [35], and it can inhibit IL-1*β*-induced inflammatory mediators in cells [36]. Moreover, myricetin was found to be the potential inhibitor of COX-2 enzyme [37]. Therefore, both flavonoids could explain, at least in part, the protective effects observed in this study regarding murine sepsis.

## 4. Conclusion

In conclusion, we have clearly demonstrated that HE-Bu inhibits important proinflammatory parameters: lethality induced by sepsis, neutrophil influx, proinflammatory cytokines, and iNOS and COX-2 expression. These beneficial effects may be related to the presence of glycosides of quercetin and myricetin in HE-Bu. This work may lead to the confirmation of the pharmacological properties of *E. uniflora* and their chemical components. Furthermore, it could also suggest a new product that, after confirming properties by clinical studies, can be used as an adjuvant in the treatment of sepsis, without the pretense of replacing the resources used in the clinic to date.

## Figures and Tables

**Table 1:** Identification of compounds detected by UHPLC-MS.

Peak	R*t *	Ion (−)	*Main fragments	Identification
1	3.58	631	479, 316, 287, 169	Myricetin-hexosyl-gallate
2	3.89	479	316, 287, 271	Myricetin-hexoside
3	3.93	463	300, 271, 255	Quercetin-hexoside
4	4.05	615	463, 300, 271, 169	Quercetin-hexosyl-gallate
5	4.20	615	463, 300, 271, 169	Quercetin-hexosyl-gallate
6	4.31	449	316, 287, 271	Myricetin-arabinoside
7	4.40	463	316, 287, 271	Myricetin-rhamnoside
8	4.55	599	447, 300, 285, 169, 124	Quercetin-rhamnosyl-gallate
9	4.70	653	501, 463, 317, 169, 124	n.i.
10	4.91	539	169, 151, 123	n.i.
11	5.05	433	301, 271, 255	Quercetin-arabinoside
12	5.28	447	301, 271, 255	Quercetin-rhamnoside
13	6.11	609	463, 300, 271	Quercetin-rhamnosyl-hexoside

*Negative fragment-ions.

n.i.: not identified.

## Abbreviations

CLP: Cecal ligation and puncture
HE-Bu: Flavonoid-rich fraction
TNF-*α*: Tumor necrosis factor-*α*
IL-1*β*: Interleukin-1*β*
NO: Nitric oxide
iNOS: Inducible nitric oxide synthase
COX-2: Cyclooxygenase 2
MPO: Myeloperoxidase
UHPLC-MS: Ultrahigh performance liquid chromatography-mass spectrometry
GC-MS: Gas chromatography-mass spectrometry.
